# Effect of Hydrogen Electrosorption on Mechanical and Electronic Properties of Pd_80_Rh_20_ Alloy

**DOI:** 10.3390/ma13010162

**Published:** 2020-01-01

**Authors:** Bozena Losiewicz, Julian Kubisztal, Patrycja Osak, Oliwia Starczewska

**Affiliations:** Institute of Materials Engineering, Faculty of Science and Technology, University of Silesia in Katowice, 75 Pulku Piechoty 1A, 41-500 Chorzow, Poland; julian.kubisztal@us.edu.pl (J.K.); patrycja.osak@us.edu.pl (P.O.); oliwia.starczewska@us.edu.pl (O.S.)

**Keywords:** hydrogen electrosorption, metallic hydride, mechanical properties, microstructure, PdRh alloy, work function

## Abstract

The interaction of hydrogen with Pt-group metals and alloys is at the center of research in the fields of electrochemistry, electrocatalysis, hydrogen technologies and fuel cells developed under the Hydrogen Economy. In this work, the material under study was Pd_80_Rh_20_ alloy (50 μm foil) subjected to hydrogen electrosorption at potentials corresponding to formation of α, α-β and β phase in 0.1 M H_2_SO_4_ at 25 °C. The total amount of hydrogen adsorbed at the surface and absorbed in octahedral interstitial positions of fcc Pd_80_Rh_20_ alloy, was determined from the oxidation charges. The H/(Pd+Rh) was 0.002, 0.4 and 0.8 for α, α-β, and β Pd_80_Rh_20_H, respectively. Microindentation hardness testing and nanoindentation showed weakening of mechanical properties of the Pd_80_Rh_20_ alloy after hydrogen electrosorption due to internal stresses. Decrease of work function with increasing amount of hydrogen absorbed occurred due to the surface roughness changes and the presence of electropositive hydrogen atoms absorbed in the crystal lattice responsible for the dipole interaction. The detailed mechanism of hydrogen absorption/diffusion in the Pd_80_Rh_20_ alloy structure is discussed. The obtained results give a new insight into the relationship between the amount of absorbed hydrogen and mechanical and electronic properties of the Pd_80_Rh_20_ alloy at the micro- and nanoscale.

## 1. Introduction

The current need for economic and scientific progress in the field of hydrogen technologies is enormous. Global failures in the broad commercialization of existing fuel cell technologies are associated with the implementation of technologies based on materials technologically underdeveloped. Reversible hydrogen storage for stationary applications based on established technologies of compressed, liquid or slush hydrogen is no longer a special problem, but the satisfactory results for hydrogen storage in containers intended for vehicles using hydrogen as fuel have still not been achieved [1,2].

Hydrogen is very reactive and forms a hydride phase or can be dissolved in a solid solution with many metals and alloys. Therefore, intensive research has been carried out on the safe storage of hydrogen in the crystal structures of metals and their alloys [1,3]. Conventional metallic hydrides (MHs) have been well characterized and there is reliable information about interstitial hydrogen storage. Many proposed MHs for use in hydrogen technologies may be formed by electrochemical reactions, providing high safety and high hydrogen storage densities. However, their application is still limited, as they are capable of storing hydrogen in insufficient quantities for fuel cell-powered electric cars [3]. Most of the research focuses on the optimization of MHs for the best practical performance; however, too little effort has been made towards investigating the effect of stored hydrogen on the structure, mechanical properties and chemical stability of MHs [2]. Interaction of hydrogen in MHs is an extremely complex phenomenon. The degree of degradation of mechanical properties is considered to be the basic criterion for the hydrogen destruction of MHs. A key issue for assessing the susceptibility of a material to hydrogen destruction is to determine the relationship between the mechanical parameters and the quantitative description of the hydrogen interaction in the microstructure. 

It has been shown that of all metals, palladium absorbs hydrogen most easily at room temperature, forming palladium hydride, which is a nonstoichiometric compound, PdH*_x_* [4,5,6]. Pd is also characterized by considerable hydrogen permeability and is called a metal sponge because it can absorb up to 900 times its own volume of hydrogen at standard temperature and pressure. The absorption of hydrogen into Pd from the gas phase [7] and under electrochemical conditions [6,8,9,10,11,12,13,14,15] at ambient temperature is reversible and leads to formation of two different crystalline phases, α and β. The α phase is a solid solution of hydrogen in the host metal, and the β phase is a metallic hydride formed with increasing hydrogen content. Under electrochemical conditions, the limit of hydrogen absorption is PdH_0.65_ [8]. It means that hydrogen occupies about 65% of the octahedral sites in a face-centered cubic (fcc) crystal structure of Pd. References [4,5,6] are comprehensive studies on the subject of the palladium-hydrogen system, and their contribution to the work concerns many important aspects. The mechanism, kinetics and thermodynamic parameters of hydrogen sorption in pure Pd electrode in the form of thin Pd films on polycrystalline Au substrate [8,9,10,13,14], thin Pd layers on Au(111) [11], Pd/Pt(111) multilayers [12], Pd monolayers [15], micrometric foil [8,9], and membranes [8,9] have been studied. The above-mentioned features of Pd have been used in the design of efficient and safe storage media for hydrogen fuel. The role and importance of Pd in Hydrogen Economy has been emphasized [16]. On the other hand, the high cost and great atomic mass limit the use of Pd for the needs of hydrogen energy, and are the reasons for searching for ways of convenient alloying of Pd in order to expand the possibilities of its applications in the industry [17,18,19]. Pd alloyed with other transition metals like Ni, Co, Cu, Zr and other has been proposed for the hydrogen sensors [20,21]. Various single, binary or ternary Pd alloys have been reported as potential candidate materials for next-generation gas sensors. Taking into account the high tolerance towards toxic H_2_S gas, ternary Pd alloys in particular are promising materials for applications in the chemical and food industries [20]. Pd and its alloys have also been intensively studied for hydrogen detection and hydrogen-related catalytic reactions, even in extremely cold environments (e.g., liquid hydrogen tanks and pipes, cryogenic hydrogen fuel in rockets) and in much warmer devices (membranes, fuel cells) [21]. 

It has been reported that larger amounts of hydrogen can be absorbed in PdRh alloys with Rh ≤ 20 at.% than in Pd [22,23,24,25,26,27,28,29,30]. It is interesting that Rh as a non-absorbing alloying addition increases the ability to hydrogen absorption in the β phase in comparison to pure Pd. The presence of Rh in PdRh alloys increases the number of the 4d band vacancies facilitating the interaction with hydrogen whose electrons are transferred to this band [23]. In our earlier study [31], we found that the kinetics of hydrogen adsorption on polycrystalline Rh in sulfuric and perchloric solutions was slower than that on Pd [8,10,11,12,13,14,15]. For the PdRhH systems containing from 20 to 30 at.% Rh in the gas phase under high H_2_ pressures, the hydrogen-to-metal ratio H/(Pd+Rh)_max_ is close to 1.0, and the separation of hydride phase is possible [28]. Under electrochemical conditions, the H/(Pd+Rh)_max_ for the Pd_80_Rh_20_H system after cathodic hydriding is 0.90 and 0.95 in acidic and alkaline solutions, respectively [23]. Slightly lower values of the H/(Pd+Rh) were determined after hydrogen absorption in 0.5 M H_2_SO_4_ on Pd_80_Rh_20_ limited-volume electrodes (LVEs), but higher when compared to that for pure Pd LVE [25]. In our previous study of the Pd_80_Rh_20_ alloy, we revealed that hydrogen adsorption inhibited the kinetics of hydrogen absorption and proceeded much faster in the presence of crystal violet added into 0.1 M H_2_SO_4_ [29]. Based on electrochemical impedance spectroscopy (EIS) measurements, it was ascertained that hydrogen electrosorption influenced the weakening of corrosion resistance of the Pd_80_Rh_20_ alloy in acidic solution. It was observed that the local impedance at corrosion potential was lower than the bulk impedance under comparable potential conditions due to the presence of internal stresses and microcracks [30]. In this report, we continue our interest in practical aspects concerning the influence of hydrogen electrosorption on mechanical and electronic properties of the Pd_80_Rh_20_ alloy in the form of 50 µm foil at the micro- and nanoscale.

## 2. Materials and Methods

### 2.1. Material Preparation

The studied samples of Pd_80_Rh_20_ (at.%) foil of a size 25 × 25 mm (±1 mm) were prepared by arc-melting the constituent elements of minimum purity 99.95% for platinum and 99.9% for rhodium (Goodfellow, Coraopolis, PA, USA). The ingot was then annealed to ensure complete mixing and rolled to the thickness *l* = 50 µm (±5 µm). Before experiments, Pd_80_Rh_20_ foil was annealed to remove stress at 650 °C for 3 h at a heating rate of 10 °C min^−1^ under the ultra-high purity argon (Ar UHP 5.0, Praxair Canada Inc., Mississauga, ON, Canada) followed by slow cooling at a rate of 0.5 °C min^−1^ to ambient temperature. The desired thickness of the Pd_80_Rh_20_ foil (50 μm) was obtained by rolling. Prior to the research, homogeneous annealing was used to reduce the heterogeneity of the chemical composition of the alloy in which undesirable microsegregation could occur and remove the stresses introduced during rolling. Stress removal was carried out in a furnace with a protective gas to prevent oxidation of the alloy surface. After annealing, the foil was slowly cooled in the furnace.

### 2.2. Electrochemical Measurement Conditions

Electrochemical measurements were carried out in 0.1 M H_2_SO_4_ solution deaerated by bubbling the ultrahigh purity argon (Ar UHP 5.0, Praxair Canada Inc., Mississauga, ON, Canada) for 30 min. During all experiments, Ar was circulated above the solution. The H_2_SO_4_ reagent of the highest purity available (Aldrich, 99.999%, St. Louis, MO, USA) and ultra-pure water (Millipore, resistivity of 18.2 MΩ cm at 25 °C, Burlington, MA, USA) were used.

A two-compartment electrochemical cell with a three-electrode system was applied. The working electrode (WE) was the Pd_80_Rh_20_ foil with a geometric surface area, *A*g, of 0.873 cm^2^ attached to an annealed gold wire. Before the measurements, the WE was pre-cleaned by immersion in 0.1 M H_2_SO_4_ solution for 15 s and application of a cathodic current of 1 mA cm^−2^ for 50 s. A pure Pt (99.99%) grid served as a counter electrode (CE). The Hg|Hg_2_SO_4_|0.1 M H_2_SO_4_ reference electrode (RE) was connected to the solution through the bridge filled with 0.1 M H_2_SO_4_ and a Luggin capillary. All the potentials are in reference to the RHE in the same solution and expressed as overpotentials. The equilibrium potential (*E*_eq_) of the RHE versus Hg|Hg_2_SO_4_|0.1 M H_2_SO_4_ electrode was −715 mV. All measurements were conducted at room temperature.

The electrochemical investigations started with recording of cyclic voltammograms in the potential range from 1.37 to 0.07 V at 20 mV s^−1^. After confirming the cleanliness of the WE surface, EIS measurements were performed at the double layer potential of *E*_dl_ = 515 mV using a Solartron 1254 Frequency Response Analyzer and PAR potentiostat/galvanostat model 273A. Ten frequencies per decade were scanned in the frequency range from 10 kHz to 1 Hz applying ac amplitude of 5 mV. The EIS data were analyzed using the complex non-linear least squares (CNLS) method with calculated modulus and allowed to determine the real surface area of the WE [32]. The statistical importance of the fit parameters was confirmed using F-test for the addition of a new parameter in the equivalent electrical circuit.

Hydrogen electrosorption was conducted using chronoamperometric measurements at the potential of 50, −33 and −150 mV until hydrogen absorption equilibrium conditions were reached. The values of hydrogen electrosorption potentials were selected on the basis of the hydrogen absorption isotherms determined for 50 μm Pd_80_Rh_20_ foil in 0.1 M H_2_SO_4_ [29]. Then, to quantify the amount of hydrogen absorbed into the Pd_80_Rh_20_ foil, the hydrogen oxidation was performed using a single scan at 20 mV s^−1^ from a given potential of hydrogen absorption to the *E*_dl_ = 515 mV. The latter potential was held for 30 min. This procedure was necessary for high hydrogen concentrations to oxidize all the hydrogen present in the foil [8,29].

To remove hydrogen gas evolved around the electrode in the solution at slightly positive overpotentials according to the Nernst equation, the solution was bubbled with argon during the saturation and oxidation of the electrode [33]. In this way, an increase in the total charge of hydrogen oxidation was avoided. The total amount of hydrogen adsorbed at the electrode surface and absorbed into the foil from its both sides, was determined by integration of the registered *j* = f(*t*) curves. All presented parameters were recalculated per real surface area.

### 2.3. Material Characterization Methods

Microstructure observations of the as-received Pd_80_Rh_20_ foil were carried out using a JEOL JSM-6480 scanning electron microscope (SEM, Peabody, MA, USA), equipped with an energy dispersive spectroscopy (EDS) system to measure the local chemical composition.

Mechanical properties at the microscale of the Pd_80_Rh_20_ foil before and immediately after hydrogen electrosorption were studied by microindentation hardness testing using a WOLPERT 401MVD microhardness tester (Bretzfeld, Germany). Ten indentations along each sample were made using a square-based diamond pyramid Vickers indenter. The maximum pressure was 0.1 N and the time of action of the load was 15 s. The impression length, measured microscopically, and the test load were used to calculate a microhardness value of μHV_0.1_.

A Hysitron TI 950 Triboscope nanoindenter (Bruker, Billerica, MA, USA) in conjunction with a QScope 250 atomic force microsope (AFM) was used to perform imaging, nanoindentation and nanomachining tests immediately after hydrogen electrosorption study. The indenter tip was used to image a 20 μm × 20 μm area of the Pd_80_Rh_20_ foil before and after hydrogen electrosorption, and then indent it with the same tip. The indentation impression was imaged with the same tip. The in situ scanning probe microscopy imaging capability was critical for precise test placement and microstructure identification. For pre- and post-test observation of the sample surface, the in situ images were obtained by raster scanning the nanoindenter probe over the sample surface. Position of the nanoindenter probe within 10 nm of the desired testing location was possible. Post-test imaging also allowed to verify if the nanoindentation test was conducted in the desired location. Such a technique maximized the reliability of test data and aided in the explanation of unexpected results. Hardness and elastic modulus were calculated from the load-displacement data obtained by nanoindentation. A Berkovich nanoindenter tip was used in the form of a three-sided diamond pyramid which was geometrically self-similar [34]. The nanoindenter monitored and recorded the load and displacement of the indenter during indentation with a force resolution of about 400 μN and displacement resolution of about 0.2 nm. The indentation experiment consisted of the following sequential steps: (i) approaching the surface, (ii) loading to peak load (5 s), (iii) holding the indenter at peak load for 2 s to avoid the effect of creep on the unloading characteristics, and (iv) unloading completely (5 s). Effects of thermal drifts were corrected in each test using a holding segment in air before indentation.

The scanning Kelvin probe (SKP) measurements were performed through a PAR Model 370 Scanning Electrochemical Workstation on the Pd_80_Rh_20_ foil before and immediately after hydrogen electrosorption. The distance between the probe-tip and the WE surface was approximately 90 µm. The contact potential difference (*V*_CPD_) was registered using the tungsten SKP microprobe (Model SKPR897, Uniscan Instruments, Buxton, UK) with a vibration amplitude of 30 μm in step mode over a designated area of the electrode surface 100 µm × 100 µm, with a 1 µm step length. Number of samplings at a specific point was 100 and the sampling frequency was 1000 Hz. The tip-sample system was considered as a capacitor, and the *V*_CPD_ was the difference in work function (*WF*) between sample and tip [35]:

(1) $$V_{\mathrm{CPD}}=\frac{WF_{\mathrm{sample}}-WF_{\mathrm{tip}}}{e},$$

where *e* is the elementary charge. Taking into account that desorption of stored hydrogen occurred with time at room temperature in ambient condition, the SKP measurements were carried out immediately after removing the electrode from the solution, rinsing, drying, taking about 2–3 min.

## 3. Results and Discussion

### 3.1. Microstructure Studies

**Figure 1:**
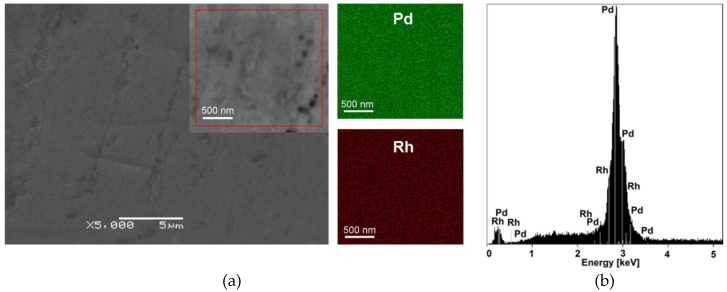
SEM image of the 50 μm Pd_80_Rh_20_ foil after annealing: (**a**) Bird’s-eye general view of the material (inset shows a detail of a selected micro-region) with the corresponding EDS map of Pd and Rh element distribution, obtained from the zoomed SEM image shown in the inset; (**b**) Energy dispersive spectrum of the Pd_80_Rh_20_ foil in the micro-region.

It is known that a decisive influence on the course of the absorption of hydrogen by metals and their alloys has the purity of both hydrogen and metal, and especially the purity of the metal surface, which can be provided by preliminary mechanical or thermal treatment, etc. Therefore, before experiments, the 50 μm Pd_80_Rh_20_ foil was subjected to the appropriate heat treatment to homogenize the chemical composition and relieve stresses introduced during the production of the material. Observations of the microstructure of the annealed Pd_80_Rh_20_ foil using SEM reveal that the surface morphology of the sample is smooth without visible microcracks even in the zoomed SEM image shown in the inset (Figure 1a); however, some subtle traces of the rolling process are present. Figure 1a also contains the corresponding maps of the distribution of chemical elements on the scanned surface (see the inset). Each map is represented in a different color, which makes it possible to extract the location of Pd (green) and Rh (red) elements in the binary alloy. The maps show that both elements are homogeneously distributed in the investigated micro-region; however, Pd is present in a greater amount. The EDS spectra were collected from different locations on a homogeneous alloy, where each single scan area was 10 μm × 10 μm. A representative energy dispersive spectrum is presented in Figure 1b, in which the characteristic peaks of the chemical constituents of Pd and Rh are present. The element concentration from the peaks obtained was determined to be 81.2 ± 0.1 at.% for Pd and 18.8 ± 0.1 at.% for Rh, which is consistent with the bulk composition of the foil under study.

### 3.2. Quantitative Study of Hydrogen Electrosorption

**Figure 2:**
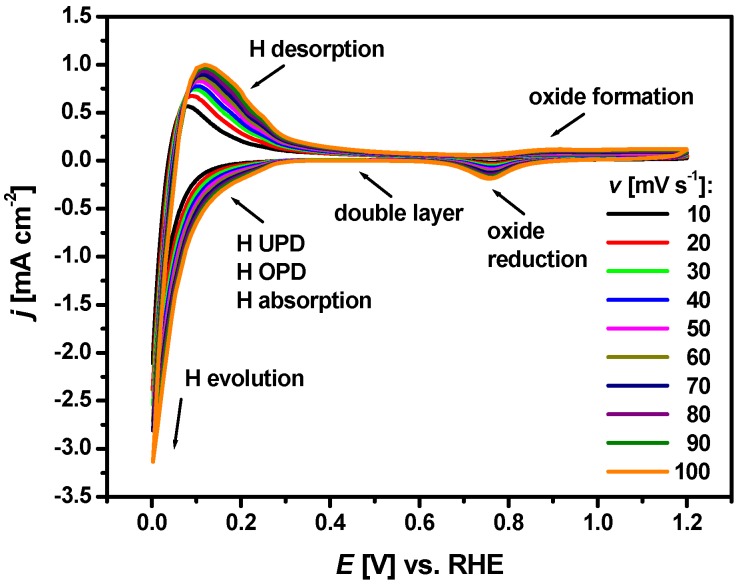
Cyclic voltammograms in the hydrogen and oxygen regions at *v* = 10–100 mV s^−1^ at 50 μm Pd_80_Rh_20_ electrode in 0.1 M H_2_SO_4_ at 25 °C.

Prior to hydrogen electrosorption, the cleanliness of the WE surface was controlled by recording of cyclic voltammograms in the hydrogen and oxygen regions at a sweep rate of 10–100 mV s^−1^ in 0.1 M H_2_SO_4_, as shown in Figure 2. In the cathodic part, one can observe the well-developed zone of the double layer, followed by a very poorly developed peak at positive potentials associated with hydrogen adsorption/absorption processes.

The absorption of hydrogen into the Pd_80_Rh_20_ foil can take place in accordance with two mechanisms [29,32]:

#### 3.2.1. Two-step indirect absorption mechanism

Two-step indirect absorption mechanism where in a first step hydrogen is adsorbed at the alloy surface in the Volmer reaction:

(2a) $$H^{+}{\;+\;M\;+\;e}^{-}\;\overset{k_{1}}{\underset{k_{-1}}{⇄}}{\;\mathrm{MH}}_{\mathrm{ads}}$$

It should be noted that the hydrogen adsorbed on the alloy occurs in two independent forms as under potentially deposited hydrogen (H_UPD_):

(2b) $$H^{+}{\;+\;M\;+\;e}^{-}\;\overset{k_{1,\mathrm{UPD}}}{\underset{k_{-1,\mathrm{UPD}}}{⇄}}{\;\mathrm{MH}}_{\mathrm{ads},\mathrm{UPD}}$$

and over potentially deposited hydrogen (H_OPD_) [29,32,36]:

(2c) $$H^{+}{\;+\;M\;+\;e}^{-}\;\overset{k_{1},\mathrm{OPD}}{\underset{k_{-1},\mathrm{OPD}}{⇄}}{\;\mathrm{MH}}_{\mathrm{ads},\mathrm{OPD}}$$

The H_OPD_ appears at less positive potentials just before the hydrogen equilibrium potential and takes part in the hydrogen evolution reaction (HER). Both H_UPD_ and H_OPD_ may participate in hydrogen absorption. The second step is described by the equation below, and is characterized by absorption of hydrogen into the alloy:

(3) $${\mathrm{MH}}_{\mathrm{ads},\mathrm{UPD}\;\mathrm{or}\;\mathrm{OPD}}+M_{\mathrm{subsurface}}\overset{k_{4}}{\underset{k_{-4}}{⇄}}M_{\mathrm{surface}}{+\;\mathrm{MH}}_{\mathrm{abs}}{}_{,0}$$

where MH_ads_ is hydrogen atom (UPD or OPD) adsorbed on the alloy surface, and MH_abs,0_ is hydrogen absorbed in the subsurface layer.

The H OPD adsorption reaction may be followed by the HER via the Heyrovský:

(4) $${\mathrm{MH}}_{\mathrm{ads},\mathrm{OPD}}+H^{+}+e^{-}\overset{k_{2}}{\underset{k_{-2}}{⇄}}{M+\;H}_{2}↑$$

or the Tafel reaction:

(5) $${2\mathrm{MH}}_{\mathrm{ads},\mathrm{OPD}}\overset{k_{3}}{\underset{k_{-3}}{⇄}}{2M+\;H}_{2}↑$$

#### 3.2.2. One-step indirect absorption mechanism

A one-step direct absorption mechanism in which hydrogen ion is reduced and absorbed in the same step [11,29,32,37,38,39,40]:

(6) $$H^{+}{+\;M\;+\;e}^{-}\overset{k_{6}}{\underset{k_{-6}}{⇄}}{\;\mathrm{MH}}_{\mathrm{abs},0}$$

Similar voltammetric behavior is also characteristic for monometals such as Pd [8,9,10,11,12,13,14,15], Rh [31], Pt [10,11,41], Ru [42], and Ir [43], where hydrogen adsorption takes place at potentials more positive than the hydrogen evolution equilibrium potential. This phenomenon called hydrogen underpotential deposition (H_UPD_) indicates strong interaction between hydrogen and these Pt-group metals. In Figure 2, the processes of hydrogen adsorption, absorption and evolution are not separated. In the anodic part of the hydrogen region, a peak due to hydrogen desorption accompanied by bisulfate adsorption reaction [31] is visible. The current densities related to the volume process of hydrogen absorption/desorption are high and they completely mask the small current densities associated with surface processes of hydrogen adsorption and oxidation. In such cases, direct determination of the amount of absorbed hydrogen into thick Pd_80_Rh_20_ foil is impossible. At more positive potentials in the oxygen region, a well-defined wave due to oxide formation is present in the anodic part. The oxide reduction peak in the cathodic scan does not overlap with the H adsorption/absorption zone as is the case with pure Rh [31]. The shape of the cyclic voltammogram at 50 μm Pd_80_Rh_20_ foil is similar to that of 50 μm Pd foil under comparable electrochemical conditions [8,29]. However, Rh as an alloying addition causes shift of the cyclic voltammogram towards negative potentials, which is characteristic for contracted alloys with a lower lattice parameter than pure Pd [25,28]. 

**Figure 3:**
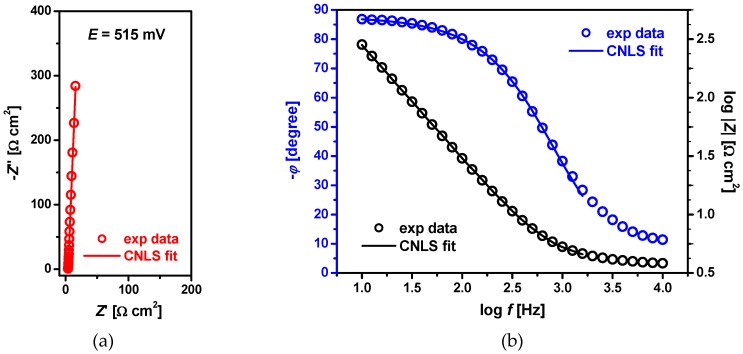
Experimental (exp data) and simulated (CNLS fit) Bode diagrams recorded at 50 μm Pd_80_Rh_20_ electrode at E = 515 mV in 0.1 M H_2_SO_4_ at 25 °C: (**a**) −Z” vs. Z’; (**b**) −ϕ vs. log f and log |Z| vs. log f. The parameters of the CNLS approximations: R_s_ = 3.51 ± 0.02 Ω cm^2^, ϕ = 0.978 ± 0.001°, and T = 48.92 ± 2.61 μF cm^−2^ s^ϕ−1^.

The real surface area of 50 μm Pd_80_Rh_20_ electrode in 0.1 M H_2_SO_4_ before hydrogen electrosorption was determined based on EIS measurements registered in the reflective mode at *E* = 515 mV. The example of the experimental complex plane plot (symbol) shows an almost vertical, capacitive line at the whole range of frequencies (Figure 3a). The corresponding Bode plots are presented in Figure 3b. The *ϕ* parameter of the the constant phase element (CPE) indicates that its deviation from the ideal capacitive behavior is very small (Figure 3b). The studied interface was described by a simple *R_s_*-CPE connection in series, where *R_s_* is the solution resistance and CPE is associated with the double layer capacitance, *C_dl_* [44]. The parameters of the CNLS approximation with their standard deviations are presented with Figure 3. The calculated on their base average ${\bar{C}}_{\mathrm{dl}}$ value was 50.1 ± 0.3 μF cm^−2^. To determine the real surface area of the Pd_80_Rh_20_ electrode the assumption was made that ultrathin films of Pd electrodeposited on Au(111) single crystal are smooth with ${\bar{C}}_{\mathrm{dl}}$ of 24.5 μF cm^−2^, which leads to a roughness factor of *R_f_* = 2 [11]. 

**Figure 4:**
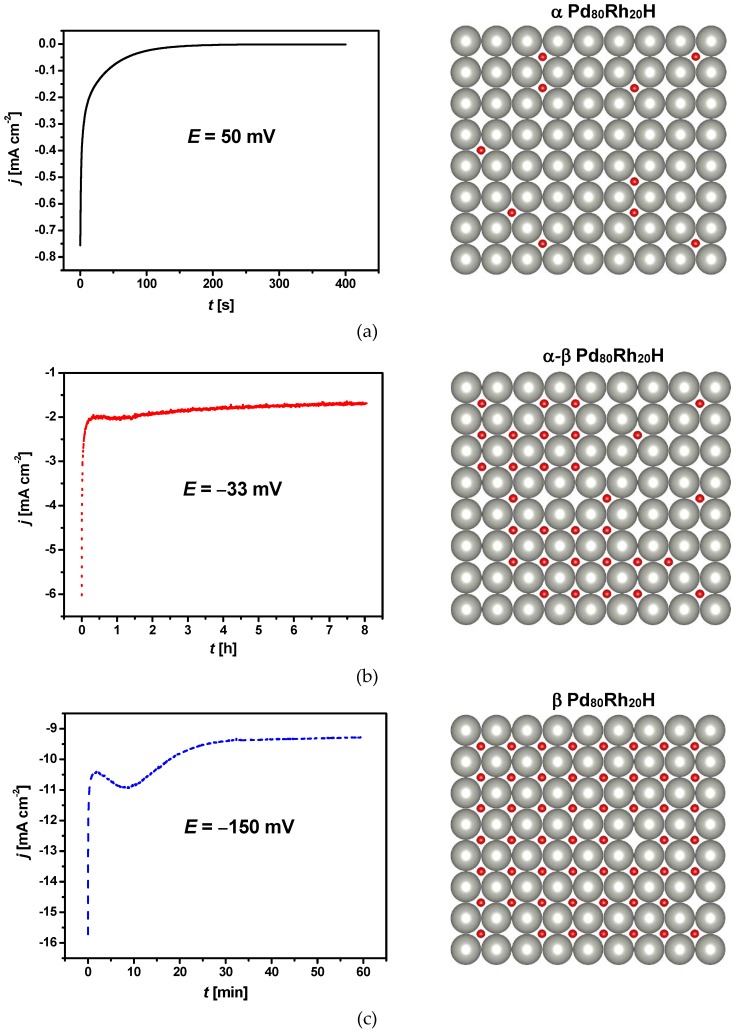
Chronoamperometric curves for hydrogen electrosorption, registered at a 50 μm Pd_80_Rh_20_ foil in 0.1M H_2_SO_4_ at different potentials corresponding to: (**a**) α phase; (**b**) α-β phase transition; (**c**) β phase. The absorbed hydrogen atoms in the corresponding structures are depicted in red.

Hydrogen electrosorption into Pd-rich (≥ 80% Pd) PdRh alloy layers electrodeposited on a gold substrate has been studied previously in 0.5 M H_2_SO_4_ using chronoamperometry and cyclic voltammetry [25]. The amount of absorbed hydrogen into such LVEs was strongly dependent on the electrode potential increasing towards negative potentials. The absorption of hydrogen at ambient temperature and pressure produced two different phases, α-phase with a low atomic ratio H/(Pd+Rh), and β-phase with H/(Pd+Rh) at high concentrations. The α-β phase transition potential was more negative than that for pure Pd. Based on those results and our own previous investigations of hydrogen absorption into Pd_80_Rh_20_ foil in 0.1 M H_2_SO_4_, the chronoamperometric conditions of hydrogen electrosorption were selected as corresponding to the formation of α Pd_80_Rh_20_H (E = 50 mV), α-β Pd_80_Rh_20_H (E = −33 mV) and β Pd_80_Rh_20_H (E = −150 mV) [29]. Figure 4 shows the chronoamperometric curves obtained at different potentials corresponding to α phase, α-β phase transition, and β phase into 50 μm Pd_80_Rh_20_ foil in 0.1 M H_2_SO_4_ at 25 °C. The corresponding structures of the α, α-β and β Pd_80_Rh_20_H are also presented. The saturation equilibrium at E = 50 mV was attained the fastest after only 250 s (Figure 4a). The α Pd_80_Rh_20_H formed in such a short time is the solid solution of hydrogen in the alloy containing low concentration of hydrogen. The longest time of ~7 h was required for complete hydrogen charging at E = −33 mV where the α-β phase transition occurred (Figure 4b). Within the α-β Pd_80_Rh_20_H, both the metal-hydrogen solution and the hydride phase coexist. At E = −150 mV hydrogen charging was completed after ~35 min (Figure 4c). The formed β Pd_80_Rh_20_H is the metallic hydride containing a large concentration of hydrogen. Moreover, the effect of HER can be expected at negative potentials. In the chronoamperometric curves, Figure 4, a constant current related to HER is observed after reaching equilibrium, whose value increases towards negative potentials.

**Figure 5:**
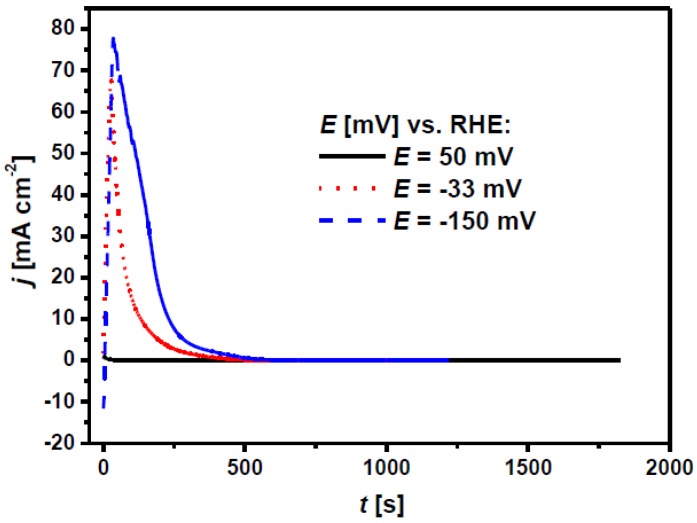
Hydrogen oxidation currents after hydrogen electrosorption at potentials corresponding to formation of α phase (*E* = 50 mV), α-β phase transition (*E* = −33 mV) and β phase (*E* = −150 mV) at 50 μm Pd_80_Rh_20_ electrode in 0.1 M H_2_SO_4_ at 25 °C.

Figure 5 presents the oxidation current densities of hydrogen absorbed at different potentials corresponding to α, β and α-β phase transition into 50 μm Pd_80_Rh_20_ foil in 0.1 M H_2_SO_4_ at 25 °C. Both height and peak charge increase with potential change towards negative potential. The total amount of hydrogen adsorbed on and absorbed into Pd_80_Rh_20_ foil, H/(Pd+Rh), was determined from the oxidation charges converted to the number of hydrogen moles, *n*_H_, and the ratio, *n*_H/(Pd+Rh)_, defined as the total number of reoxidized hydrogen moles divided by the number of Pd moles, *n*_Pd_, and the number of Rh moles, *n*_Rh_, in the foil under study [29]:

(7) $$\frac{H}{\mathrm{Pd}+\mathrm{Rh}}=n_{H/(\mathrm{Pd}+\mathrm{Rh})}=\frac{n_{H}}{n_{\mathrm{Pd}}+n_{\mathrm{Rh}}}$$

The determined value of H/(Pd+Rh) was 0.002 for α Pd_80_Rh_20_H, 0.4 for α-β Pd_80_Rh_20_H, and 0.8 for β Pd_80_Rh_20_H. 

**Figure 6:**
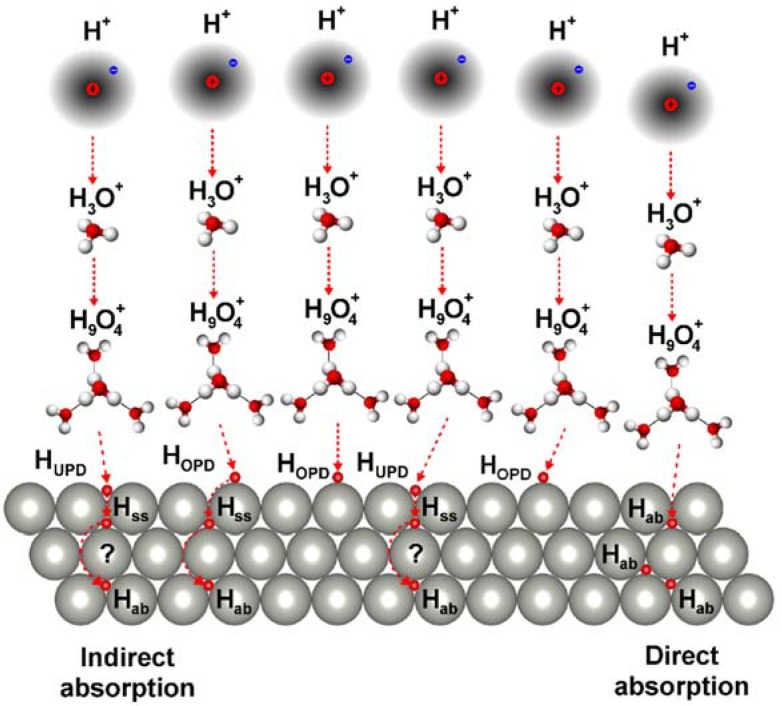
A schematic model of hydrogen absorption mechanism into Pd_80_Rh_20_ foil of the fcc structure in aqueous acidic solution in which the hydrogen atoms are depicted in red.

Figure 6 presents schematically the possible mechanisms of electrochemical absorption of hydrogen into Pd_80_Rh_20_ foil of the fcc structure in aqueous acidic solution. Close-packed lattice allows the maximum amount of interaction between atoms and leads to more energetically stable structures widely seen in metallic, atomic, and simple ionic crystals. Hexagonal packing of a single layer is more efficient than square-packing, and is considered in the following discussion.

A dissociated acid ensures the presence of a source of protons. The term proton refers to the positively charged hydrogen ion (H^+^) that is hydrated by water molecules. A recent review on the structure and dynamics of the hydrogen ion in water is available [45]. In aqueous solution, hydrogen bonds between water molecules are continually formed and broken. Water is easily protonated and deprotonated from acid, forming in initial hydration the hydroxonium ion (H_3_O^+^) with a flattened trigonal pyramidal structure, as shown in Figure 6. An effective ionic radius of the H_3_O^+^ is 0.100 nm, and it is slightly smaller than the radius of the H_2_O molecule equal to 0.138 nm [46]. Further hydration of the H_3_O^+^ ion occurs by attaching three molecules of H_2_O that are connected by hydrogen bonds, forming the hydrated hydroxonium ion (H_9_O_4_^+^), also known as an Eigen cation [47]. The H_9_O_4_^+^ is slightly more stable than other hydrated proton species in aqueous solution due to electronic delocalization over several molecules of H_2_O being preferred over the nuclear delocalization, and therefore it is more common in acidic solutions. The Eigen cation structure has the H_3_O^+^ at the center of an H_9_O_4_^+^ complex in which the hydroxonium ion interacts much more strongly with the oxygen of three neighboring molecules of H_2_O than with a weakly forming hydrogen bonds [48]. After external application of the hydrogen electrosorption potential to the positive and negative ends of electrodes immersed in an acidic electrolyte, the ordered motion of the charged ions in the electric field succeeds, and a direct current begins to flow between electrodes. The hydrogen ions move toward the cathode made of the Pd_80_Rh_20_ alloy, whereas hydroxide and other anions move toward the anode. A diaphragm should be used for separation of the cationic and the anionic electrolytes. Electrons are consumed at the cathode surface by the H_9_O_4_^+^ complex for discharging with formation of the electroadsorbed hydrogen atom; Equation (2a). Hydrogen adatom can be bonded in one of two possible forms of H_UPD_, Equation (2b), or H_OPD_, Equation (2c). The water molecules from the destroyed hydration shell can react with the electrode surface and other soluble species in the electrolyte. There is no consensus as to the adsorption sites occupied by the H_UPD_ and H_OPD_ adatoms. Most studies indicate that the H_UPD_ occupies multifold surface adsorption sites and the H_OPD_ mono-coordinated (on top) sites [46,49]. However, the suggestion that the H_UPD_ adatoms occupy the top surface adsorption sites has also appeared. Based on the thermodynamic results, it can be concluded that the M-H_UPD_ bond is very strong, and the H_UPD_ adatoms occupy multi-fold adsorption sites. However, it is not known whether multifold hollow sites, coplanar sites, or subsurface sites are preferred [46]. In the case of the fcc crystal structure, two types of threefold hollow sites are available: octahedral and tetrahedral sites. Considering that binding energy of the octahedral site is slightly larger than that of the tetrahedral site, and taking into account the fact that the H_UPD_ adatom is bonded in the threefold hollow site, the octahedral site is preferred due to its greater binding energy [46,50]. The H_OPD_ adatom is an intermediate of HER, leading to saturation of the electrolyte with dissolved hydrogen, while the H_UPD_ adatom does not participate in this process. The HER can proceed via the Volmer-Heyrovský mechanism according to Equations (2c) and (4), or the Volmer-Tafel mechanism (only in limited potential range) described by Equations (2c) and (5). Also Volmer-Heyrovský-Tafel mechanism described by Equations (2c), (4) and (5) is possible. Both the H_UPD_ and H_OPD_ adatoms can be transferred to subsurface sites. Then, the subsurface hydrogen (H_SS_) becomes absorbed hydrogen (H_ab_) according to Equation (6), and undergoes diffusion into bulk lattice sites to form a M-H solid solution referred as α, α-β or β-phase depending on the potential of hydrogen electrosorption. The hydrogen diffusion process is driven by concentration gradient of the H_ab_ [46]. It is not clear whether the two forms of adsorbed hydrogen involved in the absorption reaction, or only the H_OPD_. In metal hydrides of the fcc structure, hydrogen atoms occupy the octahedral interstitial sites in closest packing, which can accommodate additional smaller atoms or ions. The absorbed hydrogen atom is surrounded by six metal atoms forming an octahedron. The dissolution of large quantities of hydrogen atoms causes an expansion of the host metal lattice of 2 to 3 Å^3^ per hydrogen atom and hydrogen-induced internal stresses. The interatomic interactions are strained that the Pd-Rh bonds are compressed, as noted by Raub [51] and Flanagan et al. [52]. 

Hydrogen absorption into Pd_80_Rh_20_ foil in aqueous acidic solution can take place according to the two-step indirect absorption mechanism or/and the one-step direct absorption mechanism [29]. The kinetics of hydrogen electroadsorption is relatively slow, as determined on 1 ML Pd in cyclic voltammetry and EIS measurements [11]. Therefore, this reaction is considered to be the step limiting the overall rate of the indirect hydrogen absorption. These two paths of hydrogen absorption with different kinetics can coexist, where the fast path is the direct absorption mechanism observed practically, and the much slower path is the indirect mechanism. In the case of hydrogen absorption into Pd and its alloys from a gas phase, only the indirect absorption mechanism involving the hydrogen adsorption step is observed.

### 3.3. Microindentation Hardness Testing

**Figure 7:**
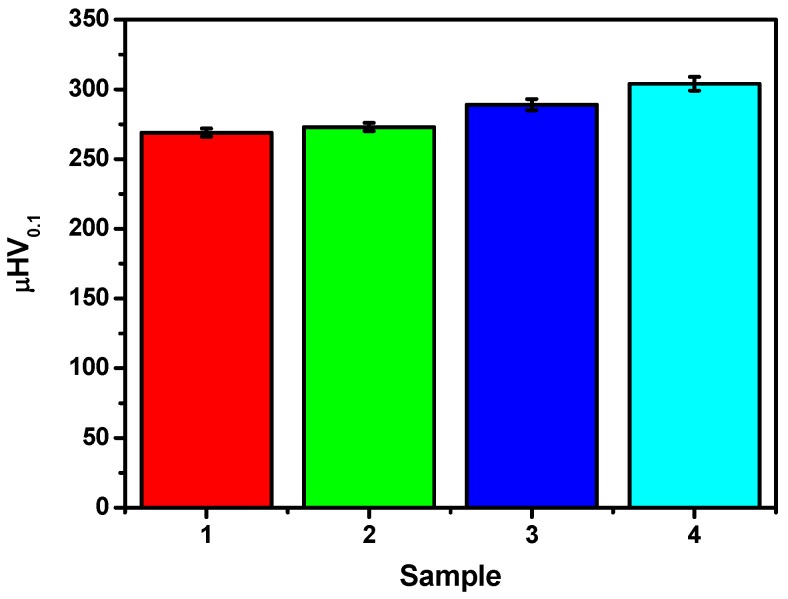
A Vickers microindentation on a surface of the Pd_80_Rh_20_ alloy (1), α Pd_80_Rh_20_H where H/(Pd+Rh) is 0.002 (2), α-β Pd_80_Rh_20_H where H/(Pd+Rh) is 0.4 (3), and β Pd_80_Rh_20_H where H/(Pd+Rh) is 0.8 (4).

The effect of hydrogen electrosorption on the mechanical properties of the Pd_80_Rh_20_ foil was studied by microindentation hardness testing. The results of a Vickers microhardness test on a surface of the Pd_80_Rh_20_ alloy before and after electrosorption are presented in Figure 7. 

The microhardness measurements reveal changes in the value of µHV_0.1_ after hydrogen absorption into the Pd_80_Rh_20_ alloy. The increase in the µHV_0.1_ with increasing content of absorbed hydrogen indicates worsening micromechanical properties. The largest microhardness is observed for β Pd_80_Rh_20_H for which µHV_0.1_ is 304 ± 5 when H/(Pd+Rh) is 0.8 (see Figure 5). The results of the hardness measurements obtained on a microscopic scale are useful indicators of the material’s properties and expected service behavior.

### 3.4. Nanoindentation

**Figure 8:**
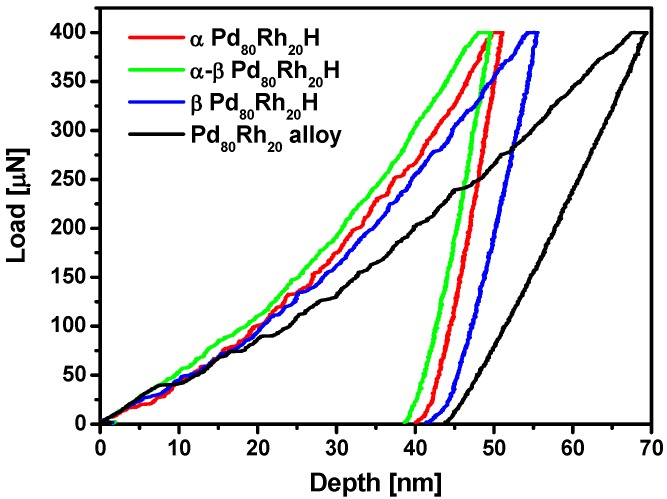
A comparison of representative nanoindentation load-displacement curves for the Pd_80_Rh_20_ alloy, α Pd_80_Rh_20_H where H/(Pd+Rh) is 0.002, α-β Pd_80_Rh_20_H where H/(Pd+Rh) is 0.4, and β Pd_80_Rh_20_H where H/(Pd+Rh) is 0.8.

The mechanical properties of the Pd_80_Rh_20_ foil before and after hydrogen electrosorption were evaluated at the nanoscale by direct nanomechanical machining using a nanoindenter and an AFM. Figure 8 shows a comparison of nanoindentation load-displacement response for the as-received Pd_80_Rh_20_ alloy, and α, α-β, and β Pd_80_Rh_20_H. A decrease of indentation depth (size) for the Pd_80_Rh_20_ alloy after hydrogen electrosorption was found, indicating that the nanomechanical properties had been weakened. Creep was observed at the peak indentation of 400 μN during the holding segment. Such behavior is observed for soft materials where dislocations have probably moved beneath the indenter tip during the indentation holding segment, contributing to an increase in indentation depth [53].

**Figure 9:**
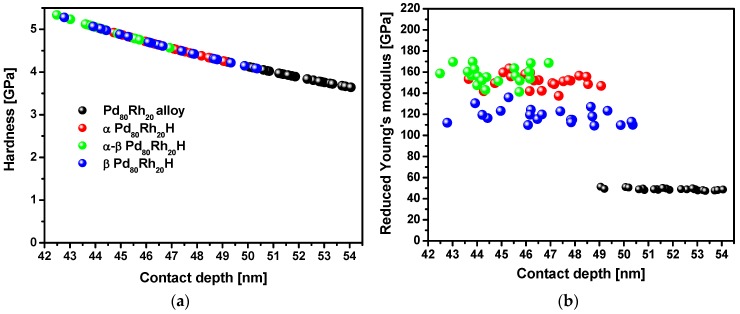
Distribution of nanomechanical parameters for the Pd_80_Rh_20_ alloy, α Pd_80_Rh_20_H, α-β Pd_80_Rh_20_H, and β Pd_80_Rh_20_H as a function of the contact depth: (**a**) nanohardness; (**b**) reduced Young’s modulus. Symbols in (**a**) and (**b**) are the same.

Distribution of nanohardness, *H*, and reduced Young’s modulus, *E*_r_, relative to the contact depth is shown in Figure 9a,b, respectively. The values of *H* and *E*_r_ for the investigated samples are shown in Table 1. The Pd_80_Rh_20_ alloy exhibits lower hardness and elastic modulus than α, α-β and β Pd_80_Rh_20_H. It is believed that the higher *H* and *E*_r_ of the Pd_80_Rh_20_ foil after hydrogen electrosorption result from the presence of hydrogen absorbed in octahedral interstitial positions leading to internal stresses of the material. At the corners of the indent, no cracks were observed.

**Figure 10:**
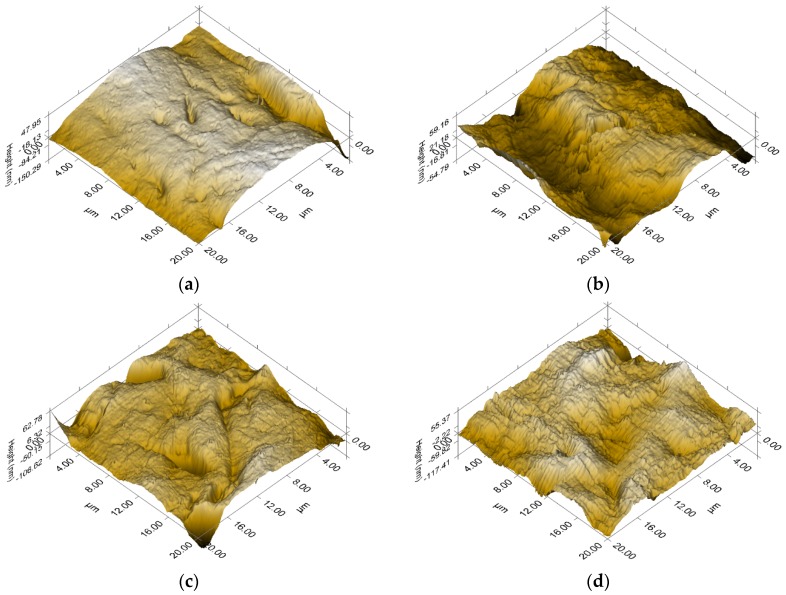
Topography SPM for the Pd_80_Rh_20_ alloy before and after hydrogen electrosorption: (**a**) Pd_80_Rh_20_ alloy; (**b**) α Pd_80_Rh_20_H, (**c**) α-β Pd_80_Rh_20_H; (**d**) β Pd_80_Rh_20_H.

Figure 10 presents the AFM images of the surface topography of the material under study, on the basis of which a two-dimensional roughness parameter, *R*a, was estimated (Table 1). The qualitative and quantitative surface topography demonstrated different degrees of roughness. The complexity of the surface topography increases with increasing amounts of absorbed hydrogen. It can be observed that the smooth surface of the Pd_80_Rh_20_ alloy shows a smaller value of *R*a, amounting 65.76, than the group of samples after hydrogen electrosorption (Figure 10a). For the non-destructive α phase of Pd_80_Rh_20_H, almost three-fold increase in *R*a is demonstrated (Figure 10b). The surface topography of the α-β phase of Pd_80_Rh_20_H is characterized by flat areas mingled with multidirectional grooves with sharp edges, and shows *R*a of 115.02 (Figure 10c). In the group of the Pd_80_Rh_20_H, the largest surface development with *R*a, amounting 276.95, is observed for the destructive β phase. Its surface topography is undulating, with a homogeneous distribution of numerous high peaks and deep valleys (Figure 10d), suggesting that absorbed hydrogen strongly modulates the surface morphology. 

Based on the studies conducted, it can be stated that hydrogen absorption into the Pd_80_Rh_20_ alloy significantly alters its nanomechanical properties increasing the value of nanohardness, reducing the elasticity and increasing the roughness. Controlled change of these parameters with a good understanding of the electrochemical process can have significant application use.

### 3.5. Determination of Work Function

**Figure 11:**
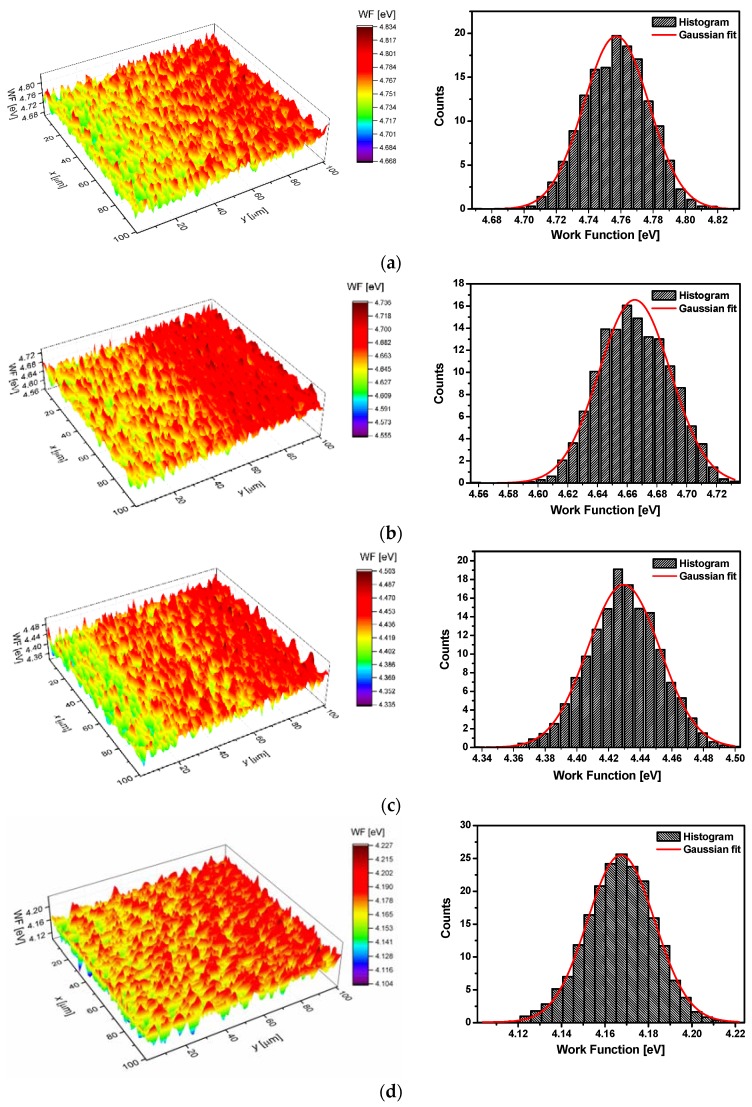
A surface distribution map of the work function for the Pd_80_Rh_20_ alloy before and after hydrogen electrosorption with the corresponding work function distribution histogram and Gaussian fitting curve: (**a**) Pd_80_Rh_20_ alloy; (**b**) α Pd_80_Rh_20_H; (**c**) α-β Pd_80_Rh_20_H; (**d**) β Pd_80_Rh_20_H.

The effect of hydrogen absorption into the Pd_80_Rh_20_ alloy on work function (*WF*) was determined with the use of the contact potential difference (*V*_CPD_) by the SKP method. The *WF* is the most fundamental electronic property of a metallic surface, and its knowledge plays an important role in understanding many surface phenomena. The *WF* is not a characteristic of a bulk material, but rather a property of the surface of the material depending on factors which change the surface dipole moment. Therefore, the *WF* is a function of the position of the Fermi level, i.e., the type of metal, the state and structure of the surface, temperature, contamination, defects, and the type and thickness of adsorbate. This is a macroscopic value, usually of several eV [54]. Surface distribution maps of the *WF* for the Pd_80_Rh_20_ foil before and after hydrogen electrosorption are presented in Figure 11. 

These maps (*z* variable) were used to obtain histograms. Each histogram was created by dividing the range of work function into 30 equal intervals (Δ*W*) and by determining the value of the work function *n_i_* lying in the range of each interval. In this way, the function *ρ*(*W_i_*) can be approximated by *n_i_*/Σ*_i_n_i_*Δ*W*. In addition, assuming that the interval Δ*W* is small enough, we approximated *ρ*(*z_i_*) by a continuous function *ρ*(*z*), and for this function we assumed a Gaussian form. The obtained histograms were fitted by Gaussian function in the following form:

(8) $$y=\frac{A}{\sigma \sqrt{2\pi }}e^{-\frac{{\left(x-\bar{x}\right)}^{2}}{2\sigma^{2}}},$$

where *A* is a constant (equal to 1); *σ* is the standard deviation; $\bar{x}$ is the average value of the expectation of Gaussian distribution, meaning the location of the peak value of the *WF*; and *σ*^2^ is the variance of Gaussian distribution, meaning the measure of the width of the *WF* distribution. Gaussian distribution curves of the *WF* for the Pd_80_Rh_20_ alloy and α, α-β, and β Pd_80_Rh_20_H are demonstrated in Figure 11. The obtained values of fitted parameters are presented in Table 2. 

One can observe that the value of $\bar{x}$ for the binary Pd_80_Rh_20_ alloy is 4.76. This value is slightly lower than the *WF* of 5.22 eV for a polycrystalline Pd and 4.98 eV for a polycrystalline Rh [41]. The value of $\bar{x}$ decreases as the amount of hydrogen absorbed in the Pd_80_Rh_20_ alloy increases, which means that lower and lower values of minimum energy are required to extract an electron from the surface of the α, α-β and β phase of Pd_80_Rh_20_H (Table 2). This is probably due to the surface roughness (see Figure 10).

Onuferko et al. [55] demonstrated that the *WF* from a roughened surface was less than from a smooth surface. The dipole interaction is responsible for the dependence of the work function on the type of surface. Part of the charge from the dipole layer pours into the deep places on the roughened surface, and the size reduction of the dipole layer follows, resulting in a lowering of the *WF*. Moreover, the hydrogen atoms absorbed in the crystal lattice are electropositive, leading to generation of the dipoles in a direction opposite to the direction of the double layer dipoles. Hence, the reduction of the double layer is observed, which reduces the work function. The values of *σ*^2^ are relatively small, suggesting that the *WF* is concentrated around a certain value, and varies only slightly over the surface (Table 2).

## 4. Conclusions

Hydrogen electrosorption into Pd_80_Rh_20_ alloy (50 μm foil) was studied under reflective conditions using cyclic voltammetry, electrochemical impedance spectroscopy, and chronoamperometric measurements at potentials corresponding to formation of α phase, α-β phase transition, and β phase in 0.1 M H_2_SO_4_ at 25 °C. Based on the oxidation current densities of the hydrogen absorbed, the value of H/(Pd+Rh) was determined to be 0.002 for α Pd_80_Rh_20_H, 0.4 for α-β Pd_80_Rh_20_H, and 0.8 for β Pd_80_Rh_20_H. The following mechanisms of electrochemical absorption of hydrogen into Pd_80_Rh_20_ foil in aqueous acidic solution were proposed: (i) two-step indirect absorption mechanism, where during the first step, hydrogen is adsorbed onto the alloy surface in two independent forms as under potentially deposited hydrogen and over potentially deposited hydrogen, both of which may participate in the hydrogen absorption in the second step, and (ii) one-step direct absorption mechanism in which hydrogen ions are reduced and absorbed in the same step.

The influence of hydrogen absorption on the mechanical properties of the Pd_80_Rh_20_ alloy was ascertained by microindentation hardness testing and nanoindentation. The results of a Vickers microhardness test indicated worsening micromechanical properties with increasing content of absorbed hydrogen. The nanoindentation results for the Pd_80_Rh_20_ alloy exhibit lower hardness and elastic modulus than those for α, α-β and β Pd_80_Rh_20_H due to the hydrogen absorbed in octahedral interstitial positions, leading to internal stresses of the material.

The effect of hydrogen absorption into the Pd_80_Rh_20_ alloy on work function was determined through the use of the contact potential difference by the SKP method. The *WF* value decreases as the amount of hydrogen absorbed in the Pd_80_Rh_20_ alloy increases, due to the surface roughness changes and the presence of electropositive hydrogen atoms absorbed in the crystal lattice being responsible for the dipole interaction.

## Figures and Tables

**Table 1:** Nanohardness, reduced Young’s modulus, and average roughness values for the Pd_80_Rh_20_ alloy and α, α-β, β Pd_80_Rh_20_H.

No.	Sample	Nanohardness *H* [GPa]	Reduced Young’s Modulus *E*r [GPa]	Average Roughness *R*a [nm]
1	Pd_80_Rh_20_ alloy	3.90 ± 0.18	49.04 ± 1.01	65.76
2	α Pd_80_Rh_20_H	4.64 ± 0.24	152.49 ± 8.58	192.89
3	α-β Pd_80_Rh_20_H	4.79 ± 0.17	157.66 ± 5.75	115.02
4	β Pd_80_Rh_20_H	4.57 ± 0.34	118.82 ± 7.19	276.95

**Table 2:** Values of parameters from the Gaussian distribution curves of work function for the Pd_80_Rh_20_ alloy and α, α-β, β Pd_80_Rh_20_H.

No.	Sample	$\bar{x}$	*σ* ^2^
1	Pd_80_Rh_20_H alloy	4.76	(0.02)^2^
2	α Pd_80_Rh_20_H	4.66	(0.02)^2^
3	α-β Pd_80_Rh_20_H	4.43	(0.02)^2^
4	β Pd_80_Rh_20_H	4.17	(0.02)^2^

## References

[1] Ball M., Basile A., Veziroğlu T.N. (2016). Compendium of Hydrogen Energy.

[2] Moliner R., Lázaro M.J., Suelves I. (2016). Analysis of the strategies for bridging the gap towards the hydrogen economy. Int. J. Hydrog. Energ..

[3] Rusman N.A.A., Dahari M. (2016). A review on the current progress of metal hydrides material for solid-state hydrogen. Int. J. Hydrog. Energ..

[4] Lewis F.A. (1967). The Palladium Hydrogen System.

[5] Lewis F.A. (1995). The palladium-hydrogen system: structures near phase transition and critical points. Int. J. Hydrog. Energ.

[6] Łukaszewski M., Czerwiński A. (2011). The method of limited volume electrodes as a tool for hydrogen electrosorption studies in palladium and its alloys. J. Solid State Electrochem..

[7] Yamauchi M., Ikeda R., Kitagawa H., Takata M. (2008). Nanosize effects on hydrogen storage in palladium. J. Phys. Chem. C.

[8] Birry L., Lasia A. (2006). Effect of crystal violet on the kinetics of H sorption into Pd. Electrochim. Acta.

[9] Łosiewicz B., Birry L., Lasia A. (2007). Effect of adsorbed carbon monoxide on the kinetics of hydrogen electrosorption into palladium. J. Electroanal. Chem..

[10] Jurczakowski R., Łosiewicz B., Lasia A. (2007). Kinetic and thermodynamic parameters of hydrogen sorption in Pd, Pd-Pt and on Pt. ECS Trans..

[11] Duncan H., Lasia A. (2007). Mechanism of hydrogen adsorption/absorption at thin Pd layers on Au(111). Electrochim. Acta.

[12] Duncan H., Lasia A. (2008). Hydrogen adsorption/absorption on Pd/Pt(111) multilayers. J. Electroanal. Chem..

[13] Duncan H., Lasia A. (2008). Separation of hydrogen adsorption and absorption on Pd thin films. Electrochim. Acta.

[14] Martin M.H., Lasia A. (2008). Study of the hydrogen absorption in Pd in alkaline solution. Electrochim. Acta.

[15] Martin M.H., Lasia A. (2009). Hydrogen sorption in Pd monolayers in alkaline solution. Electrochim. Acta.

[16] Adams B.D., Chen A. (2011). The role of palladium in a hydrogen economy. Mater. Today.

[17] Ouyang L., Huang J., Wang H., Liu J., Zhu M. (2017). Progress of hydrogen storage alloys for Ni-MH rechargeable power batteries in electric vehicles: A review. Mater. Chem. Phys..

[18] Hubkowska K., Łukaszewski M., Koss U., Czerwiński A. (2014). Characterization and electrochemical behavior of Pd-rich Pd-Ru alloys. Electrochim. Acta.

[19] Hubkowska K., Łukaszewski M., Czerwiński A. (2014). Thermodynamics of hydride formation and decomposition in electrodeposited Pd-rich Pd-Ru alloys. Electrochem. Commun..

[20] Sharma B., Myung J. (2019). Pd-based ternary alloys for gas sensing applications: A review. Int. J. Hydrogen Energy.

[21] Du L., Feng D., Xing X., Fu Y., Fonseca L.F., Yang D. (2019). Palladium/cobalt nanowires with improved hydrogen sensing stability at ultra-low temperatures. Nanoscale.

[22] Dos Santos D.S., Miraglia S., Fruchart D. (2004). Effects of cathodic charging on hydrogen permeation in a Pd_80_Rh_20_ alloy. J. Alloy Compd..

[23] Comisso N., De Ninno A., Del Giudice E., Mengoli G., Soldan P. (2004). Electrolytic hydriding of Pd_79.5_Rh_20.5_ alloy. Electrochim. Acta.

[24] Siwek H., Łukaszewski M., Czerwiński A. (2004). Electrosorption of carbon dioxide on Rh binary alloys with Pt and Pd. Pol. J. Chem..

[25] Żurowski A., Łukaszewski M., Czerwiński A. (2006). Electrosorption of hydrogen into palladium-rhodium alloys. Electrochim. Acta.

[26] Łukaszewski M., Żurowski A., Grdeń M., Czerwiński A. (2007). Correlations between hydrogen electrosorption properties and composition of Pd-noble metal alloys. Electrochem. Commun..

[27] Żurowski A., Łukaszewski M., Czerwiński A. (2008). Electrosorption of hydrogen into palladium-rhodium alloys; Part 2. Pd-rich electrodes of various thickness. Electrochim. Acta.

[28] Flanagan T.B., Baranowski B., Majchrzak S. (1970). Remarkable interstitial hydrogen contents observed in rhodium-palladium alloys at high pressures. J. Phys. Chem..

[29] Łosiewicz B., Lasia A. (2018). Study of the hydrogen absorption/diffusion in Pd_80_Rh_20_ alloy in acidic solution. J. Electroanal. Chem..

[30] Łosiewicz B., Kubisztal J. (2018). Effect of hydrogen electrosorption on corrosion resistance of Pd_80_Rh_20_ alloy in sulfuric acid: EIS and LEIS study. Int. J. Hydrog. Energ..

[31] Łosiewicz B., Jurczakowski R., Lasia A. (2011). Kinetics of hydrogen underpotential deposition at polycrystalline rhodium in acidic solutions. Electrochim. Acta.

[32] Lasia A. (2014). Electrochemical Impedance Spectroscopy and its Applications.

[33] Vigier F., Jurczakowski R., Lasia A. (2006). Determination of hydrogen absorption isotherm and diffusion coefficient in Pd_81_Pt_19_ alloy. J. Electroanal. Chem..

[34] Khrushchov M.M., Berkovich E.S. (1951). Methods of determining the hardness of very hard materials: The hardness of diamond. Ind. Diam. Rev..

[35] Łosiewicz B., Popczyk M., Szklarska M., Smołka A., Osak P., Budniok A. (2015). Application of the scanning Kelivin probe technique for characterization of corrosion interfaces. Solid State Phenom..

[36] Jerkiewicz G. (2010). Electrochemical hydrogen adsorption and absorption. Part 1: Under-potential deposition of hydrogen. Electrocatal..

[37] Bagotskaya I.A. (1962). Effect of the solution composition on the diffusion rate of electrolytic hydrogen. Zh. Fiz. Khim..

[38] Frumkin A.N., Delahay P.J. (1963). Advances in Electrochemistry and Electrochemical Engineering, Vol. 3: Electrochemistry.

[39] Zheng G., Popov B.N., White R.E. (1995). Hydrogen-atom direct-entry mechanism into metal membranes. J. Electrochem. Soc..

[40] Chen J.S., Diard J.P., Durand R., Montella C. (1996). Hydrogen insertion reaction with restricted diffusion. Part 1. Potential step—EIS theory and review for the direct insertion mechanism. J. Electroanal. Chem..

[41] Łosiewicz B., Jurczakowski R., Lasia A. (2012). Kinetics of hydrogen underpotential deposition at polycrystalline platinum in acidic solutions. Electrochim. Acta.

[42] Łosiewicz B., Martin M., Lebouin C., Lasia A. (2010). Kinetics of hydrogen underpotential deposition at ruthenium in acidic solutions. J. Electroanal. Chem..

[43] Łosiewicz B., Jurczakowski R., Lasia A. (2017). Kinetics of hydrogen underpotential deposition at iridium in sulfuric and perchloric acids. Electrochim. Acta.

[44] Brug G.J., Van den Eeden A.L.G., Sluyters-Rehbach M., Sluyters J.H. (1984). The analysis of electrode impedances complicated by the presence of a constant phase element. J. Electroanal. Chem..

[45] Agmon N., Bakker H.J., Campen R.K., Henchman R.H., Pohl P., Roke S., Thämer M., Hassanali A. (2016). Protons and hydroxide ions in aqueous systems. Chem. Rev..

[46] Marcus Y. (2012). Volumes of aqueous hydrogen and hydroxide ions at 0 to 200 °C. J. Chem. Phys..

[47] Kirchner B. (2007). Eigen or Zundel ion: News from calculated and experimental photoelectron spectroscopy. Chem. Phys. Chem..

[48] Mella M. (2013). Exploring unvisited regions to investigate solution properties: The backyard of H_3_O^+^ and its aggregates. Chem. Phys. Lett..

[49] Nichols R.J., Lipkowski J., Ross P.N. (1992). Adsorption of Molecules at Metal Electrodes, Chapter 7.

[50] Christman K. (1988). Interaction of hydrogen with solid surfaces. Surf. Sci. Rep..

[51] Raub E. (1959). Metals and alloys of the platinum group. J. Less-Common. Met..

[52] Flanagan T.B., Wang D., Clewley J.D., Noh H. (2000). Hydrogen solubility in ternary Pd_0.90_Rh_0.1-x_Ni_x_ and Pd_0.90_Rh_0.1-x_Co_x_ alloys. J. Alloy Comp..

[53] Li X., Nardi P., Baek C.W., Kim J.M., Kim Y.K. (2005). Direct nanomechanical machining of gold nanowires using a nanoindenter and an atomic force microscope. J. Micromech. Microeng..

[54] Haynes W.M. (2016). Handbook of Chemistry and Physics.

[55] Onuferko J.H., Woodruff D.P., Holland B.W. (1979). LEED structure analysis of the Ni{100} (2 × 2)C (p4g) structure; A case of adsorbate-induced substrate distortion. Surf. Sci..

